# Archaea: An under-estimated kingdom in livestock animals

**DOI:** 10.3389/fvets.2022.973508

**Published:** 2022-07-28

**Authors:** Yunjuan Peng, Ting Xie, Zhuosui Wu, Wenxiao Zheng, Tao Zhang, Samantha Howe, Jianmin Chai, Feilong Deng, Ying Li, Jiangchao Zhao

**Affiliations:** ^1^Guangdong Provincial Key Laboratory of Animal Molecular Design and Precise Breeding, College of Life Science and Engineering, Foshan University, Foshan, China; ^2^School of Life Science and Engineering, Foshan University, Foshan, China; ^3^Division of Agriculture, Department of Animal Science, University of Arkansas, Fayetteville, AR, United States

**Keywords:** metatranscriptome, metagenome, ruminant, pig, chicken, monogastric animals, archaea

## Abstract

Archaea are considered an essential group of gut microorganisms in both humans and animals. However, they have been neglected in previous studies, especially those involving non-ruminants. In this study, we re-analyzed published metagenomic and metatranscriptomic data sequenced from matched samples to explore the composition and the expression activity of gut archaea in ruminants (cattle and sheep) and monogastric animals (pig and chicken). Our results showed that the alpha and beta diversity of each host species, especially cattle and chickens, calculated from metagenomic and metatranscriptomic data were significantly different, suggesting that metatranscriptomic data better represent the functional status of archaea. We detected that the relative abundance of 17 (cattle), 7 (sheep), 20 (pig), and 2 (chicken) archaeal species were identified in the top 100 archaeal taxa when analyzing the metagenomic datasets, and these species were classified as the “active archaeal species” for each host species by comparison with corresponding metatranscriptomic data. For example, The expressive abundance in metatranscriptomic dataset of *Methanosphaera cuniculi* and *Methanosphaera stadtmanae* were 30- and 27-fold higher than that in metagenomic abundance, indicating their potentially important function in the pig gut. Here we aim to show the potential importance of archaea in the livestock digestive tract and encourage future research in this area, especially on the gut archaea of monogastric animals.

## Introduction

Although archaea is a relatively new domain, they are considered one of the oldest organisms on Earth. Since being described as an independent domain by Woese and Fox (1), archaea have been detected in various extreme environments, including marine environments (i.e., oceans and freshwater) and various locations in the mammalian body (2, 3).

According to 16S rRNA sequences in the SILVA database, it is estimated that 20,000 archaeal species from about 30 phyla exist worldwide (4). In the Genome Taxonomy Database (GTDB) (5, 6), a relatively complete taxonomy database of archaea and bacteria, 2339 archaeal species clusters belonging to 19 phyla have been collected from different environments based on single genomes or metagenome-assembled genomes. However, over 70% have yet to be cultured (6).

The gastrointestinal microbial community is the largest and most important ecosystem contributing to the maintenance of intestinal health in mammals, and archaea constitute an essential part of the gut microbiota of mammals. In humans, which have attracted more research attention than other mammals, archaea were detected in almost all ecological niches (7). Chibani et al. (8) assembled 1,167 archaeal genomes using globally available metagenomic datasets from the human gut, including three genera and 15 species, and suggested continued research on human gut archaea is necessary. Recently, Youngblut et al. (2) investigated gut archaeal diversity in vertebrates using archaea-targeting 16S primers and successfully amplified and sequenced eligible archaeal reads from 110 vertebrate species. Youngblut et al. (2) identified six phyla and ten archaeal classes, including four new host-associated taxa. More than 60% of the ASVs (Amplicon Sequence Variants) derived from these taxa had no matched cultured representative (similarity ≥97%), and five classes had no reference sequence with a sequence similarity of 85%, suggesting that a large knowledge gap in vertebrate gut archaea exists. Their results further revealed that host phylogeny had a stronger influence on gut archaeal diversity than diet, and some specific taxa were associated with body temperature (*Methanothermobacter*) and feeding habits (*Methanomethylophilus*). This study by Youngblut and colleagues opens new research horizons on gut archaea in vertebrates.

In ruminants, methanogens, an important subgroup of archaea, are generally considered harmful due to methane production and host energy loss. However, we believe archaea's hydrogen-consuming abilities may benefit the host, as hydrogen inhibits rumen/gut fermentation. Results from Li and Guan (9) showed that *Methanomassiliicoccales* (an archaeal genus) was significantly enriched in the rumen of beef cattle with higher feed utilization. Additionally, in another study, they identified a positive relationship between several archaeal species (such as *Methanobrevibacter smithii*) and feed utilization (10). In sheep, McLoughlin et al. (11) identified three species belonging to *Methanobrevibacter* that were significantly associated with increased feed utilization. Our recent study showed that gut archaea have the functional potential to reduce hydrogen and are involved in carbohydrate metabolism by expressing CAZyme genes in pigs (12). Therefore, the function of archaea in the digestive system of livestock is likely important and should not be overlooked. In this study, we re-analyzed published metagenomic and metatranscriptomic data sequenced from matched individuals to explore the composition and expression activity of gut archaea, providing vital information over gut archaea in livestock. We hope to promote discussion on digestive archaea and encourage future research in this area.

## Materials and methods

### Data collection and pre-processing

A total of four datasets, containing both metagenomic and metatranscriptomic sequencing reads from the rumen of cattle (*n* = 14) (10) and sheep (*n* = 10) (13) and feces from chickens (*n* = 6) (14) and pigs (*n* = 6) (14), were collected from published articles. Raw reads quality control and host-contamination filtering were performed on these collected metagenomic and metatranscriptomic data using the Kneaddata pipeline v0.7.2 (https://bitbucket.org/biobakery/kneaddata). In brief, first, raw reads were trimmed with Trimmomatic v0.39 (15). Then, host-contamination reads were identified and removed by mapping raw reads to their corresponding host reference genomes [Accession number: GCF_002263795 (Cattle), GCF_002742125 (Sheep), GCF_016699485 (Chicken) and GCF_000003025 (Pig)] with the Bowtie2 software (16). Considering the higher expression of ribosomal RNA in metatranscriptomic dataset, SortMeRNA software (v4.3.2) (17) and SMR v4.3 sensitive database were used to remove potential ribosomal RNA sequences from both metagenomic and metatranscriptomic data, to reduce the interference in quantifying expression of archaeal taxa. Clean reads were acquired for further analysis after the abovementioned raw reads processing steps.

### Archaeal taxonomy profiling and diversity calculation

For taxonomic classification of both metagenomic and metatranscriptomic data, Kraken2 version 2.1.2 (18) was used to assign clean reads to archaeal reference genomes from the Genome Taxonomy Database release 202 (GTDB 202) (19), which contained 4,316 archaeal genomes representing 2,339 archaeal species, and 245,090 bacterial genomes representing 45,555 bacterial species (Access date: October 13, 2021). The downloaded GTDB database was pre-built using the Struo2 pipeline (20). Subsequently, the metagenomic and metatranscriptomic clean reads were classified based on the GTDB database using Kraken2.

The quantitative table of archaeal and bacterial species in each sample were furtherly processed using QIIME2 platform version 2021.4 (21) to rarefy and calculate the relative abundance of bacteria and archaea in samples. A species-level rarefied archaeal reads count table was re-imported into QIIME2 to calculate archaea alpha diversity (Shannon Index) and beta diversity (Bray-Curtis).

Kruskal-Wallis test and Analysis of Similarities (ANOSIM) were performed using the QIIME2 platform. For all analyses, statistical significance was determined at *P* ≤ 0.05. All figures were generated using the R package, ggplot2 (22).

## Results and discussion

### Alpha and beta diversity

**Figure 1:**
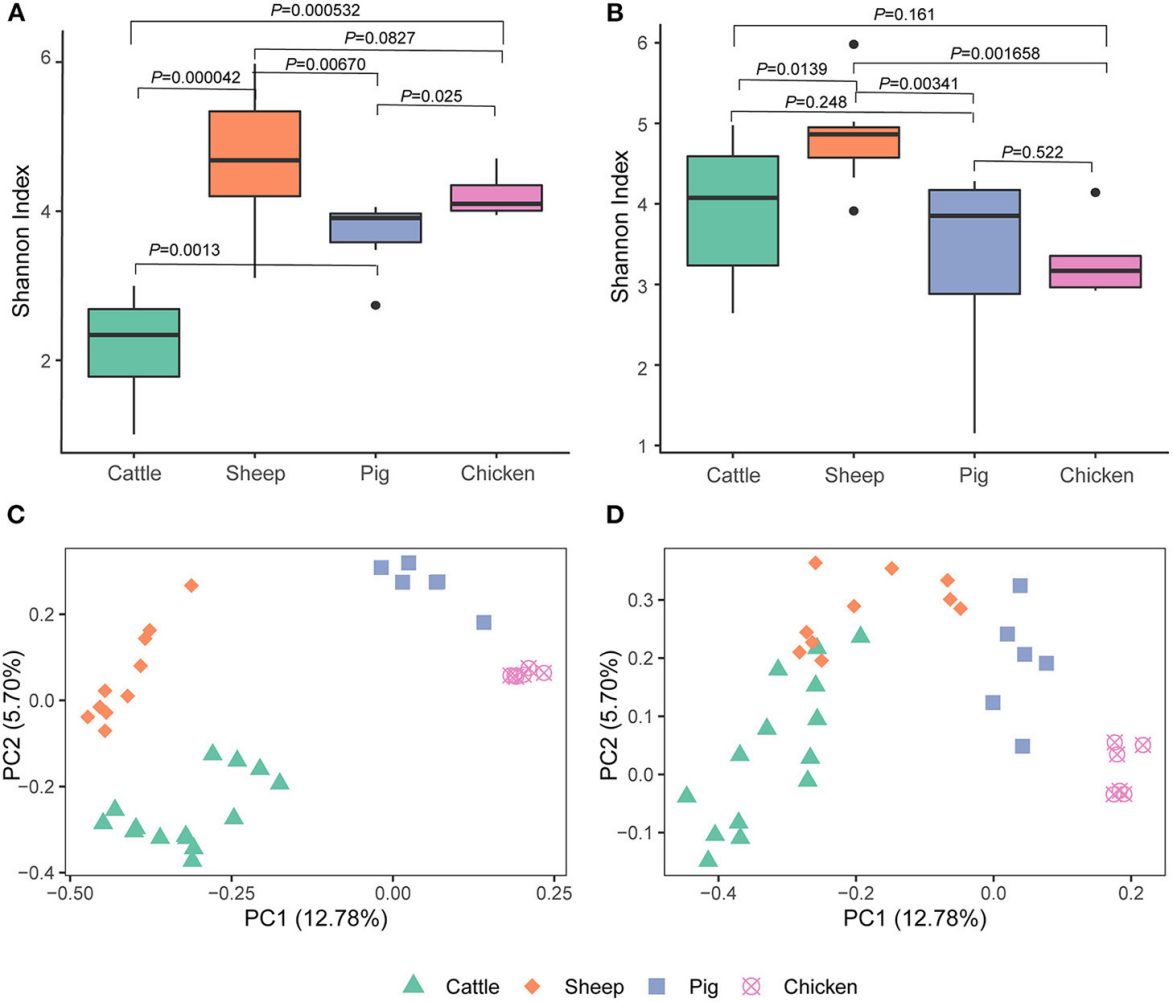
Alpha [Shannon index, **(A,B)**] and beta [Bray-Curtis, **(C,D)**] diversity of gut archaea based on the metagenome **(A,C)** and metatranscriptome **(B,D)**.

We detected differences in alpha diversity (Shannon index) among the four livestock species. Based on the metagenomic analysis, the Shannon index for cattle was significantly lower than that of sheep (cattle vs. sheep, *P* = 0.000042), pigs (cattle vs. pig, *P* = 0.0013), and chickens (cattle vs. chicken, *P* = 0.00053), while pairwise comparisons among sheep, pigs, and chickens revealed that all pairwise comparisons were at *P* > 0.01 (sheep vs. pig, *P* = 0.013; sheep vs. chicken, *P* = 0.083; chicken vs. pig, *P* = 0.025; Figure 1A). This was not the expected result, as differential alpha diversity was observed between the ruminants, and the sheep alpha diversity was more similar to that of pigs and chickens. Further analysis based on metatranscriptomic data revealed that the Shannon index of cattle almost reached that of sheep (cattle vs. sheep, *P* = 0.014). The Shannon index reflects both archaeal species richness and evenness. The higher Shannon index observed in the cattle metatranscriptome compared to the metagenome indicated that the archaeal species present had high expression activity in the cattle rumen. Additionally, no significant difference was observed between pigs (*P* = 0.248) and chickens (*P* = 0.161; Figure 1B). Moreover, the Shannon index of sheep was significantly higher than that of pigs (*P* = 0.0034) and chickens (*P* = 0.0017). No difference between chicken and pigs (*P* = 0.522) was observed.

A metagenome- and metatranscriptome- based comparison among three different cattle breeds by Li et al. (10), the results showed that a greater difference was observed between breeds at the metatranscriptomic level compared to the metagenomic level. Here, we compared the archaeal structure in livestock using both of metagenomic and metatranscriptomic sequencing data to explore the effects of the sequencing technologies on the archaeal structure. The Bray-Curtis-based PCoA plot produced using metagenomic data showed that the four livestock species were separated from each other (ANOSIM, *P* < 0.05; Figure 1C, Supplementary Table 1). Cattle and sheep were close to each other but still separate (*P* < 0.05) and were further separated from the chicken and pig clusters in the PCoA plot using metatranscriptomic dataset (Figure 1D, Supplementary Table 2).

### Disproportionally expressed archaeal species in the four livestock animals

**Figure 2:**
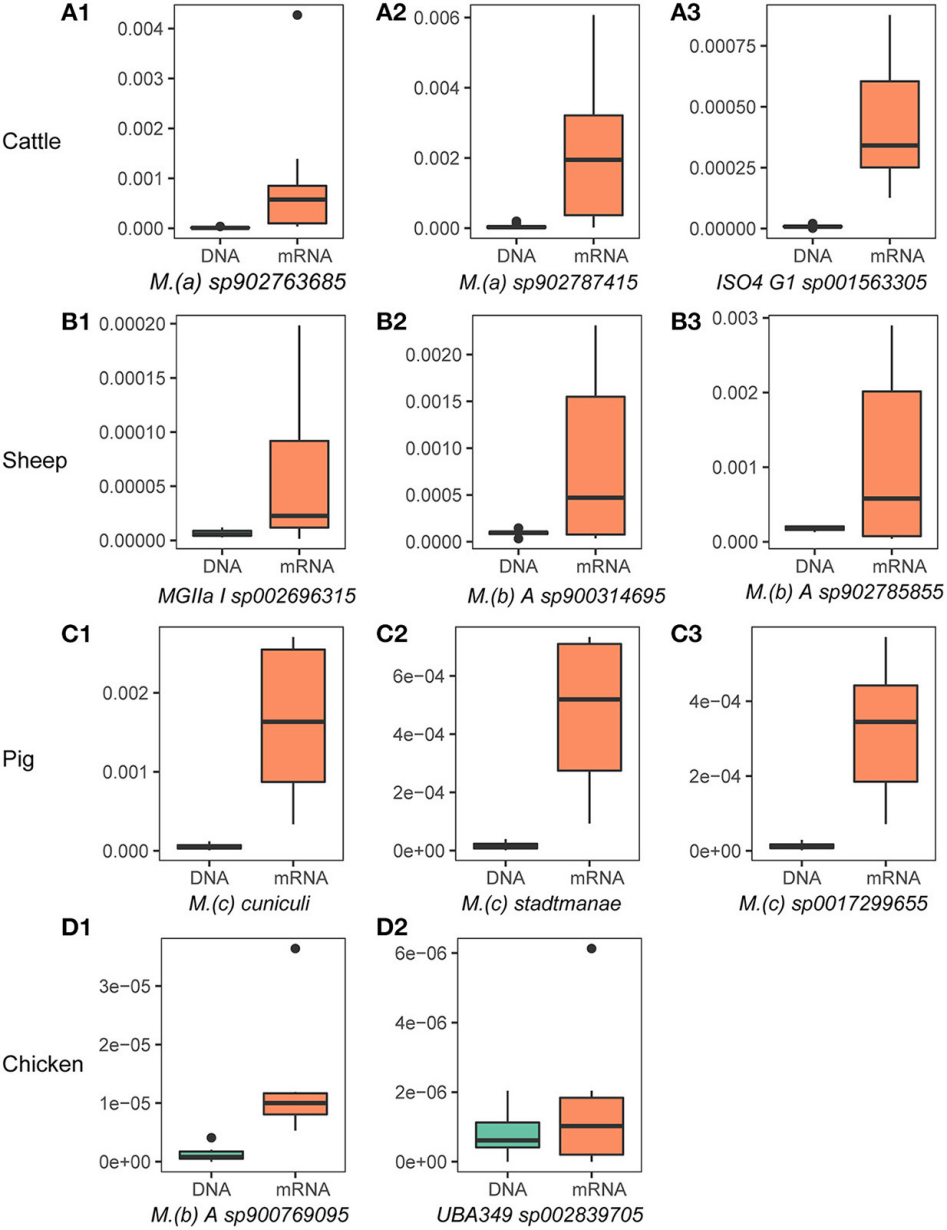
Highly active archaeal species in the rumen/gut of cattle **(A1–A3)**, sheep **(B1–B3)**, pigs **(C1–C3)**, and chickens **(D1,D2)**. The y-axis represents species' relative abundance in archaea and bacteria, and labels of DNA and mRNA on the x-axis label represent the relative abundance from metagenome and metatranscriptome, respectively. The x-axis titles are the name of archaeal species, name abbreviations of genus showed in the figure are as follows: *M.(a)*: *Methanomethylophilus, M.(b)*: *Methanobrevibacter, M.(c)*: *Methanosphaera*. ISO4 G1, MGIIa I, and *UBA349* are genus names or ids defined in the GTDB.

We further determined the “active archaeal species” by comparing the relative abundance of archaeal species in both the metagenome and metatranscriptome. We considered species to be “active archaeal species” if they (1) were observed at least in 80% of samples and (2) the mRNA relative abundance was at least 2-fold higher than the DNA relative abundance. In total, 17, 7, 20, and 2 archaeal species were identified from the top 100 relatively abundant archaeal taxa in the metagenomic dataset as “active archaeal species” for cattle, sheep, pig, and chicken, respectively (Supplementary Table 3). Figure 2 shows the highly active species identified in each host species. Of them, 6 of the 17 “active archaeal species” in cattle belong to the genus *Methanomethylophilus*, 4 of the 7 “active archaeal species” in sheep belong to genus *Methanobrevibacter*, 12 of the 20 “active archaeal species” in pig belong to the genus *Methanosphaera*, and for chicken, only two “active archaeal species” annotated as *Methanobrevibacter A sp900769095* and *UBA349 sp002839705*, suggesting archaeal taxa-specific expression activity signatures for different host species. To our surprise, archaeal species in the pig showed high expression activity. For instance, both *Methanosphaera cuniculi* (Figure 2C1) and *Methanosphaera stadtmanae* (Figure 2C2) are known as hydrogen-consuming archaea and are commonly detected in the pig gut (23), and to the best of our knowledge, no study reports solid evidence of their function. Higher expression activity (30-fold expression for *Methanosphaera cuniculi* and 27-fold expression for *Methanosphaera stadtmanae*) may indicate their important function in swine. However, they have been widely ignored in past studies due to the lower abundance of archaea.

It is thought that some archaeal taxa, especially methane-producing archaea, are key species that may affect the composition and function of the microbiota in complex host and non-host environments (24–26). In humans, some archaea, such as trimethylamine N-oxide (TMAO)-reducing archaeal species, have been believed to be potential probiotic candidates (27), while others have been linked to human diseases, such as cancer and obesity (28, 29). Taken together, accumulative evidence suggests that archaea may play important roles in host health and disease. However, the function of archaea is still greatly unknown in livestock. Some studies have focused on the negative effects of methane-producing archaea in the rumen. In addition, others have focused on the association between archaeal taxa and animal growth, identifying a link between archaeal taxa in the rumen and feed utilization (10, 11). Our recent study showed that gut archaea might promote the energy harvest in pigs due to their involvement in gut fermentation, indicating archaea may affect swine growth (12).

Samuel et al. (30) found that methanogenic archaea could reduce hydrogen, increasing host energy harvest and fat deposition in the mouse model; however, it did not seem to entirely explain the effects of methanogenic archaea on the host. Our previous study based on shotgun metagenomic data from pigs suggested that gut archaea may be directly involved in gut fermentation by expressing CAZyme genes, indicating another potential function of archaea in the gut. Archaea coexist with bacteria and fungi in different complex microbiomes. However, archaea-bacteria and archaea-fungi interactions are still poorly understood and could be another important function of archaea (31).

Until now, we have little evidence to speculate on the potential links between archaea in the digestive system and livestock growth performance. Therefore, more studies are needed to investigate the roles that archaea play in animal production.

## Conclusion

Archaea are an important component of the complex microbial ecosystem in the digestive tract of humans and animals. This study compared the effects of the metagenome and metatranscriptome on archaeal diversity and composition and revealed the difference between archaeal composition (metagenome) and expression (metatranscriptome). In addition, although the abundance of archaea in the digestive tract is relatively low, we found that the transcripts of several archaeal species are extremely high (≥2-fold), suggesting these archaea are very active and functioning in animals especially in the less-studied monogastric animals.

## Data availability statement

The original contributions presented in the study are included in the article/Supplementary material, further inquiries can be directed to the corresponding authors.

## Author contributions

YP and FD contributed to analysis, interpretation, and drafted the manuscript. YP, TX, and JC contributed to data analysis. ZW, WZ, and TZ contributed to data collection. YP, SH, JZ, and YL contributed to critically revised the manuscript. JZ and YL contributed to conception. All authors gave final approval and agreed to be accountable for all aspects of the work.

## Funding

This work was supported by National Natural Science Foundation of China (Grant No. 32170430), Guangdong Provincial Key Laboratory of Animal Molecular Design and Precise Breeding (2019B030301010), and Key Laboratory of Animal Molecular Design and Precise Breeding of Guangdong Higher Education Institutes (2019KSYS011).

## Conflict of interest

The authors declare that the research was conducted in the absence of any commercial or financial relationships that could be construed as a potential conflict of interest.

## Publisher's note

All claims expressed in this article are solely those of the authors and do not necessarily represent those of their affiliated organizations, or those of the publisher, the editors and the reviewers. Any product that may be evaluated in this article, or claim that may be made by its manufacturer, is not guaranteed or endorsed by the publisher.
